# Incidentaloma on Staging CT Revealed to Be Breast to Renal Tumour-to-Tumour Metastasis

**DOI:** 10.7759/cureus.45658

**Published:** 2023-09-21

**Authors:** Ayesha Khan, Md Abu Sayed, Khaled Hosny, Godwins Echejoh, Santhi Kumar

**Affiliations:** 1 Urology, East Lancashire Hospitals NHS Trust, Blackburn, GBR; 2 General Surgery, East Lancashire Hospitals NHS Trust, Blackburn, GBR; 3 Histopathology, East Lancashire Hospitals NHS Trust, Blackburn, GBR

**Keywords:** ct staging, incidentaloma, radical nephrectomy, tumour-to-tumour metastasis, breast cancer, renal cell carcinoma

## Abstract

Tumour-to-tumour metastases (TTM) are a rare phenomenon in which a primary tumour has metastasised within another distant primary tumour. We present the case of a 63-year-old female who presented with right-sided breast cancer. An incidental left-sided renal mass was detected on staging CT of the thorax, abdomen, and pelvis (CT-TAP). The patient had no evidence of metastases below the diaphragm. Histology following a radical left nephrectomy revealed metastatic breast cancer within a renal cell carcinoma (RCC). The patient underwent chemotherapy and surgery for right-sided breast cancer. Follow-up imaging demonstrated the metastatic spread of the breast cancer. This is an unusual case of TTM from breast to an initially occult RCC primary.

## Introduction

Tumour-to-tumour metastases (TTM) remain a rare occurrence within the biomedical literature. It is defined as the presence of a primary tumour, known as the donor tumour, within a separate primary tumour, known as the recipient [1]. Frequently reported donor tumours include lung and breast malignancy. Renal cell carcinoma (RCC) is the most common malignant recipient, which has been postulated due to its high vascularity [2]. Meningiomas have been cited as the most common benign recipient [3,4]. Breast cancer is recognised to metastasise to the lungs, bone, and brain. Renal metastases are less common, with an incidence of 12.6% in a reported autopsy series, and even lower prevalence upon clinical detection alone [5,6].

We present the case of a patient who had a left-sided renal mass incidentally detected on staging CT for a newly diagnosed right-sided breast cancer. This was suspected to be a radiologically staged T2a RCC. Following a nephrectomy, histology of the renal mass revealed RCC but with evidence of breast cancer metastasised within. 

## Case presentation

**Figure 1:**
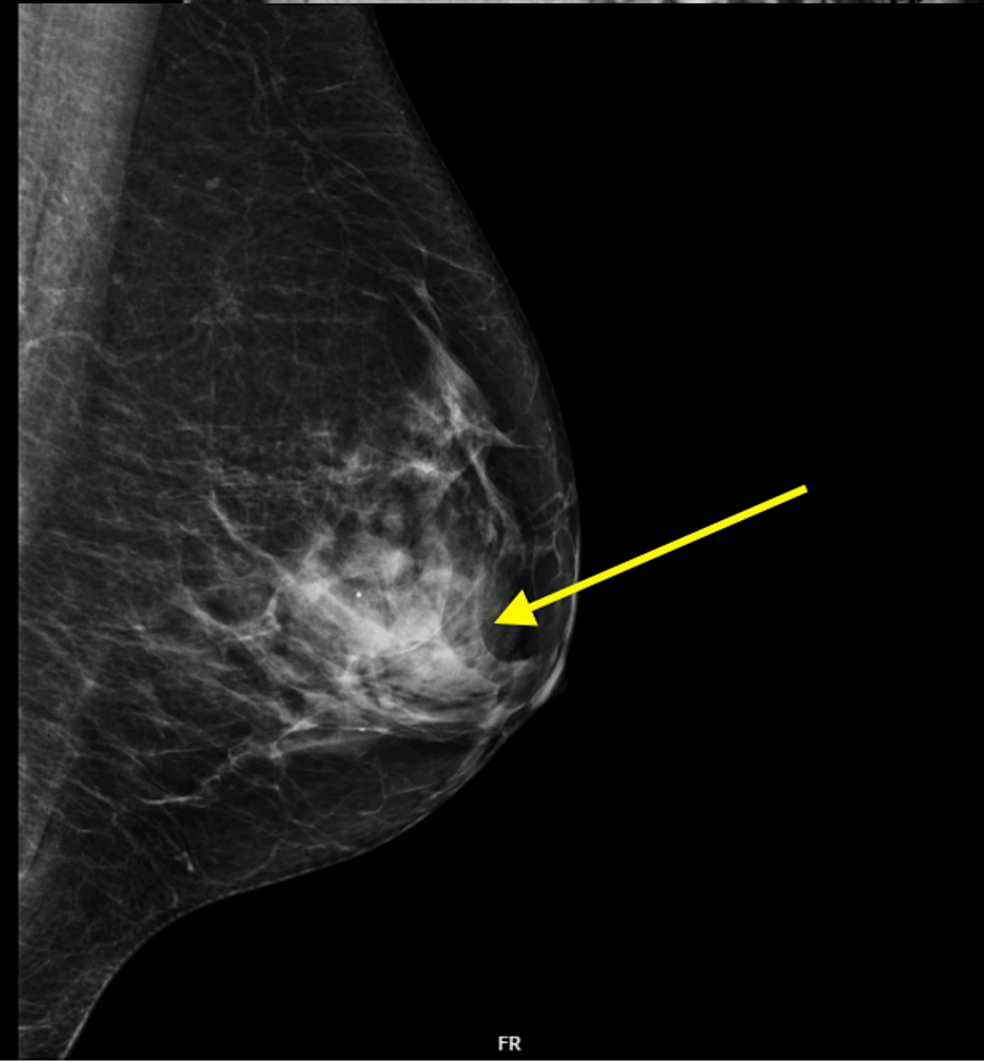
Mediolateral oblique view mammogram of the right breast showing the malignant mass.

A 62-year-old female patient attended the national breast screening programme at the end of 2021 which demonstrated a normal mammogram. Two months later, she was referred by her general practitioner (GP) to the breast clinic via the two-week wait pathway for a painless lump in her right breast. Investigation with an ultrasound scan of the right breast lump was unremarkable. Initially, the lump decreased in size but two months later it reoccurred with overlying erythema of the skin. She was started on antibiotics and subsequently reviewed in the breast clinic. On this occasion, examination of the right breast revealed significant peau d’orange (dimpling of the skin) of the medial aspect and associated erythema. A potential lymph node was also palpated in the right axilla. A mammogram of both breasts raised suspicion for a malignant mass in the right breast (Figure 1) and an ultrasound demonstrated markedly enlarged lymph nodes, suspicious for malignant involvement in the right axilla.

Biopsies of the right breast mass and suspicious malignant lymph node in the right axilla were performed. Histology of the breast mass revealed a provisional diagnosis of grade 3, invasive ductal carcinoma (IDC), oestrogen receptor (ER) negative, progesterone receptor (PR) negative and human epidermal growth factor receptor 2 (HER2) negative. Additionally, there was evidence of high-grade ductal carcinoma in situ (DCIS). There was no evidence of pleomorphic lobular carcinoma. Histology of the right axillary node confirmed malignant neoplasm favouring metastatic carcinoma. Staining revealed it was cytokeratin (CK) AE1/AE3 positive and Melan A negative.

**Figure 2:**
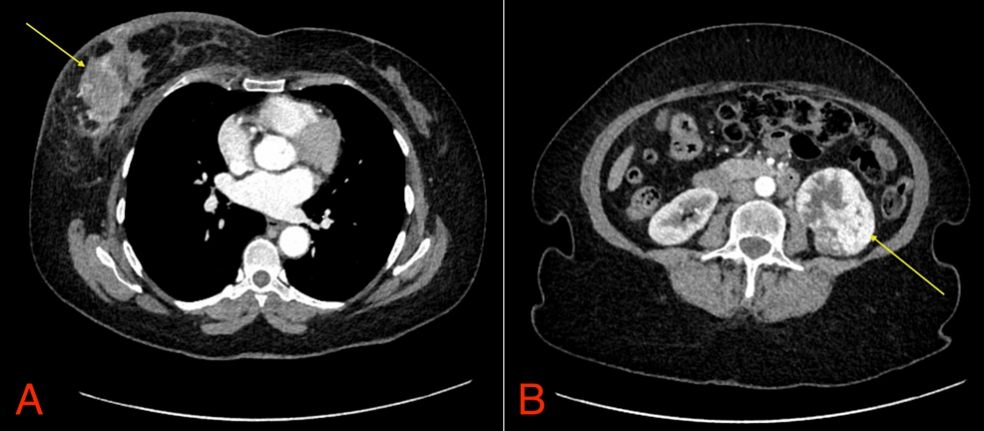
Staging CT-TAP following diagnosis of right-sided breast cancer. (A) Demonstration of right breast cancer; (B) Demonstration of left renal mass CT-TAP: CT of the thorax, abdomen, and pelvis

The patient was started on neoadjuvant epirubicin and cyclophosphamide/carboplatin/paclitaxel chemotherapy and an urgent staging CT of the thorax, abdomen, and pelvis (CT-TAP) was requested. This identified the known right breast mass (Figure 2A) and right axillary lymph nodes. It also revealed small enhancing nodes on the right pectoralis major muscle of unknown aetiology. Additionally, an incidental mass on the left kidney (Figure 2B) was detected and the patient was referred to the urology multi-disciplinary team (MDT) meeting.

After completion of neoadjuvant chemotherapy, the patient had a repeat MRI thorax to assess the chest wall abnormality and plan for surgical management. The MRI demonstrated the breast tumour abutted the pectoralis major muscle inferiorly; however, there was no clear invasion of the muscle itself. Additionally, a repeat staging CT scan showed an unchanged appearance of the breast tumour, the lymphadenopathy and the suspected left lower pole renal tumour. No other new metastatic deposits were detected elsewhere. The patient consequently underwent a right mastectomy and axillary node clearance.

Histology of the mastectomy specimen showed partial response to therapy, grade 2, IDC of no specific type, 5 mm from all margins and 1.5 mm from the skin surface. There was greater than 10% of the tumour remaining in the tumour bed seen as an area of residual fibrosis delineating the original tumour extent. The interpectoral tissue of the right breast and seven out of 17 right axillary lymph nodes demonstrated metastatic adenocarcinoma. Adjuvant radiotherapy to the chest wall and supraclavicular fossa and internal mammary chain was pursued.

**Figure 3:**
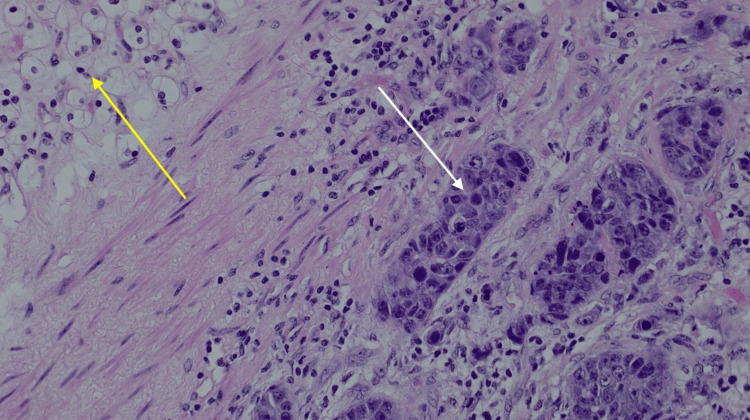
Histopathological examination. Hematoxylin and eosin stain (H&E) x20. Yellow arrow highlighting clear cell renal cell carcinoma (ccRCC) on the upper left aspect of the slide and white arrow highlighting breast cancer on the right side of the slide.

The patient was referred to the local urology centre for a left-sided radical nephrectomy. The procedure was uneventful and the patient was discharged back to her local trust for ongoing care with oncology. Histology from the nephrectomy revealed a primary renal cancer, pT3a RCC, which contained evidence of metastatic breast cancer (Figure 3). Histological analysis revealed that clear cell renal carcinoma and metastatic breast cancer invaded a tributary of the renal vein. Additionally, the tumour invaded the renal sinus fat but did not extend to invade Gerota’s fascia.

**Figure 4:**
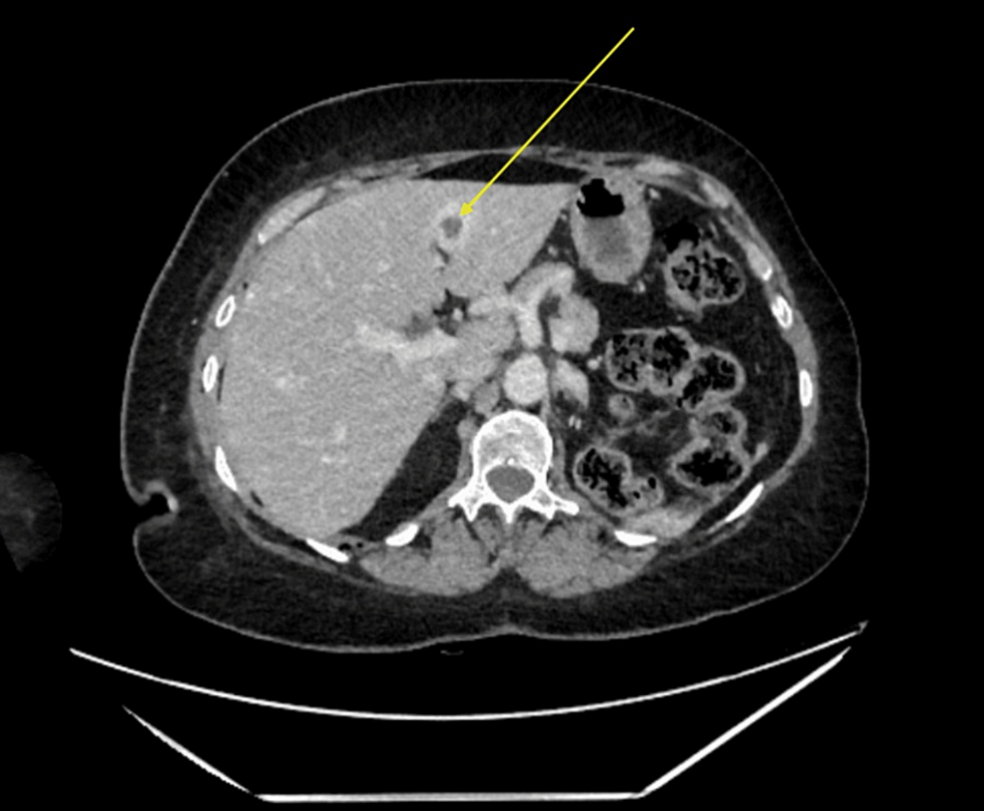
Demonstration of a liver metastasis on a follow-up CT-TAP. CT-TAP: CT of the thorax, abdomen, and pelvis (CT-TAP)

**Figure 5:**
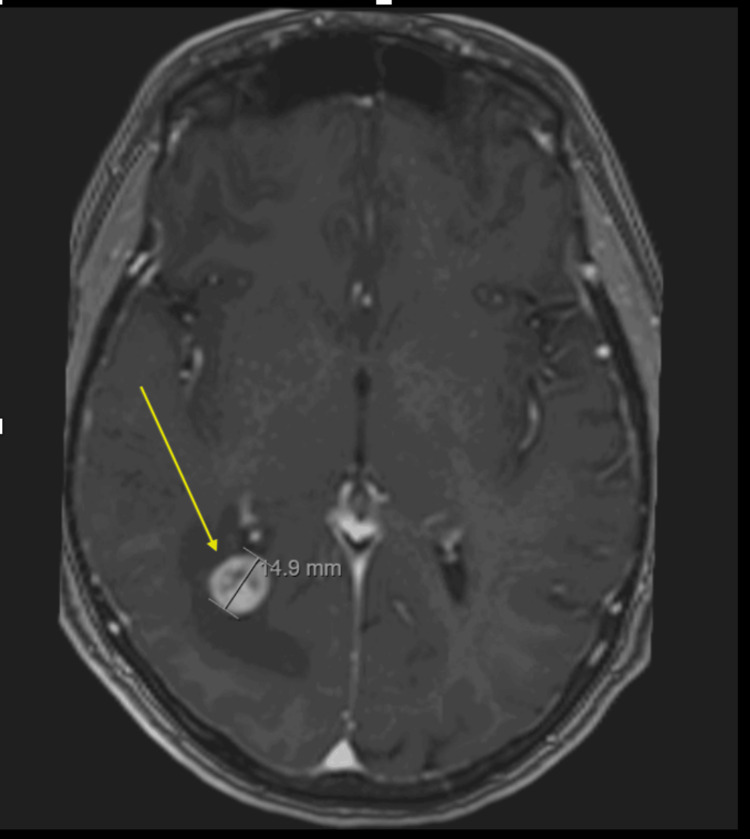
MRI head with contrast demonstrating a presumed cerebral metastasis with surrounding oedema.

The patient developed a right supraclavicular lymph node and an urgent CT revealed metastases in the axilla and liver (Figure 4) and a potential lesion in the brain, which was later confirmed on MRI head (Figure 5). The patient received stereotactic radiosurgery before repeating imaging for consideration of chemotherapy. There was no CT evidence of recurrence of the RCC. However, there was evidence of breast cancer disease progression. After completion of radiotherapy for the central nervous system (CNS) lesion, the patient was commenced on chemo-immunotherapy with an atezolizumab/nab-paclitaxel regime. The patient remains under close monitoring and management with the oncology team.

## Discussion

Breast cancer is the most commonly diagnosed cancer in women. While the majority of cases are detected on routine screening, there are a number who present to physicians with breast changes [7]. Triple-negative breast cancer is an aggressive form of breast cancer that does not display either hormone receptor (ER/PR) or HER2 (ERBB2) overexpression/amplification. This molecular subtype of breast cancer typically has a poor prognosis and high metastatic and recurrence rates due to presentation at an advanced stage [8]. Triple-negative breast cancer commonly metastasises to the lungs, brain, and liver [9]. While microscopic metastatic deposits have been detected on autopsy, radiologically detectable renal metastases are uncommon from breast cancer but have been reported [5].

RCC is the most common renal malignancy. Greater than 50% of diagnoses are made incidentally on imaging for unrelated investigations. The stage of disease on radiograph plays a crucial role in the management decisions at MDT. With early detection and management of RCC, patients have a positive prognosis [10].

Incidental findings, also known as incidentalomas, are not uncommon and may represent benign or malignant pathology. Koo et al. reported one in 25 patients with cancer were diagnosed incidentally [11]. Although still relatively uncommon, with the advances in diagnostic techniques and greater life expectancies, the detection of more than one primary malignancy in an individual has increased [12]. In our case, the patient was diagnosed with a second primary malignancy on routine staging for her breast cancer. She was otherwise asymptomatic from the RCC point of view. Histology from the nephrectomy confirmed the presence of breast cancer cells within the RCC.

TTM are a rare phenomenon. Approximately only 200 cases have been reported in the English-language biomedical literature since the first case report in 1930. In 1968, the formal four-point criteria for TTM were established by Campbell et al. [1]. The most common donor tumour in the medical literature is lung carcinoma followed by breast carcinoma [4]. Meningiomas are the most common benign recipient and renal cancers are the most common malignant tumour metastasis recipient [3]. Distant metastases may occur once cancer cells detach from the primary tumour and intravasate via the lymphatic and circulatory systems [13]. However, the exact pathogenesis of TTM is not entirely clear. In TTM, the malignant cells infiltrate and grow within a pre-existing tumour. In particular, with renal cancer as the most common recipient, one theory suggests that a greater number of viable malignant cells from the primary tumour may enter the renal tumour due to the large proportion of cardiac output arriving at the kidney [2]. Additionally, another theory proposes the environment of renal cancers is abundant in glycogen and lipid-rich cells which allows the metastases to thrive [2]. These theories help us understand why renal cancer is the most common recipient.

To our knowledge, there have been only 10 other reports of invasive ductal carcinoma of the breast metastasising to an RCC, two of which were diagnosed on autopsy [14,15]. Interestingly, from our review of the literature, this is the first case to report the TTM before any other radiologically detected metastases, besides ipsilateral axillary lymph nodes. The patient was restaged following neo-adjuvant chemotherapy and mastectomy to ensure there was no spread of disease before being referred for nephrectomy.

Metastatic breast cancer has a poor prognosis, showing a mean 26% five-year survival rate [16]. In comparison, stage one or two RCC has a significantly much favourable prognosis of 80-90% five-year survival rate [10]. In an individual with breast cancer and new renal mass, consideration is to be had regarding the nature of the renal mass in relation to the breast cancer. Renal metastases in these individuals would indicate a poor prognosis. Therefore, a nephrectomy would not be indicated.

As mentioned previously, breast cancer metastasising to the kidneys is uncommon. One established indication for image-guided renal biopsy is in patients with a concurrent extrarenal primary malignancy. Imaging of renal masses cannot distinguish between metastases and RCC with certainty as similar features can be recognised radiologically in both disease processes [17]. A renal biopsy allows for histopathological identification of the mass, thus tailoring the subsequent management plan more appropriately. Metastases are managed medically, whereas non-metastatic RCCs can be managed surgically [17]. In this case, the left renal mass was managed as a stable presumed RCC, thus the patient was listed for theatre. However, if a renal biopsy performed identified a TTM, this may have altered the patient’s management as a nephrectomy is unlikely to have improved her overall survival in light of the metastatic breast cancer. This highlights the need for consideration from urologists and radiologists at MDT for image-guided renal biopsies in the presence of an extrarenal primary cancer.

## Conclusions

We reported a case of a rare phenomenon, TTM. The patient was found to have breast cancer within an RCC. Renal malignancy is one of the most common recipients of tumour metastases and despite being a rare occurrence, urologists, radiologists, and histopathologists need to be aware of this phenomenon.

## References

[1] Campbell LV Jr, Gilbert E, Chamberlain CR Jr, Watne AL (1968). Metastases of cancer to cancer. Cancer.

[2] Möller MG, Gribbin T, Ebrom S, Padula G, Fitzgerald TL (2006). Breast cancer metastatic to renal cell carcinoma. Surgery.

[3] Sayegh ET, Burch EA, Henderson GA, Oh T, Bloch O, Parsa AT (2015). Tumor-to-tumor metastasis: breast carcinoma to meningioma. J Clin Neurosci.

[4] Talukdar A, Khanra D, Mukhopadhay S, Bose D (2014). Tumor to tumor metastasis: adenocarcinoma of lung metastatic to meningioma. J Postgrad Med.

[5] Nagata A, Shinden Y, Nomoto Y (2022). Metastasis of breast cancer to the right kidney with a tumor thrombus in the inferior vena cava: a case report. Surg Case Rep.

[6] Cazacu SM, SĂndulescu LD, Mitroi G, Neagoe DC, Streba C, Albulescu DM (2020). Metastases to the kidney: a case report and review of the literature. Curr Health Sci J.

[7] Kolak A, Kamińska M, Sygit K, Budny A, Surdyka D, Kukiełka-Budny B, Burdan F (2017). Primary and secondary prevention of breast cancer. Ann Agric Environ Med.

[8] Dass SA, Tan KL, Selva Rajan R (2021). Triple negative breast cancer: a review of present and future diagnostic modalities. Medicina (Kaunas).

[9] O'Reilly D, Sendi MA, Kelly CM (2021). Overview of recent advances in metastatic triple negative breast cancer. World J Clin Oncol.

[10] Gray RE, Harris GT (2019). Renal cell carcinoma: diagnosis and management. Am Fam Physician.

[11] Koo MM, Rubin G, McPhail S, Lyratzopoulos G (2019). Incidentally diagnosed cancer and commonly preceding clinical scenarios: a cross-sectional descriptive analysis of English audit data. BMJ Open.

[12] Jena A, Patnayak R, Lakshmi AY, Manilal B, Reddy MK (2016). Multiple primary cancers: an enigma. South Asian J Cancer.

[13] Seyfried TN, Huysentruyt LC (2013). On the origin of cancer metastasis. Crit Rev Oncog.

[14] Huo Z, Gao Y, Yu Z, Zuo W, Zhang Y (2015). Metastasis of breast cancer to renal cancer: report of a rare case. Int J Clin Exp Pathol.

[15] Lakovschek IC, Petru E, Pollheimer MJ, Ratschek M, Augustin H, Bjelic-Radisic V (2019). A rare case of cancer-to-cancer metastasis: breast cancer to renal cell cancer : case report and review of literature. Wien Med Wochenschr.

[16] Berger ER, Park T, Saridakis A, Golshan M, Greenup RA, Ahuja N (2021). Immunotherapy treatment for triple negative breast cancer. Pharmaceuticals (Basel).

[17] Sahni VA, Silverman SG (2009). Biopsy of renal masses: when and why. Cancer Imaging.

